# Advanced Wound Dressing for Real-Time pH Monitoring

**DOI:** 10.1021/acssensors.1c00552

**Published:** 2021-06-02

**Authors:** Federica Mariani, Martina Serafini, Isacco Gualandi, Danilo Arcangeli, Francesco Decataldo, Luca Possanzini, Marta Tessarolo, Domenica Tonelli, Beatrice Fraboni, Erika Scavetta

**Affiliations:** † Dipartimento di Chimica Industriale “Toso Montanari”, Università di Bologna, Viale del Risorgimento 4, 40136 Bologna, Italy; ‡ Dipartimento di Fisica e Astronomia, Università di Bologna, Viale Berti Pichat 6/2, 40127 Bologna, Italy

**Keywords:** IrOx, PEDOT:PSS, pH sensing, wound dressing, wound healing monitoring, bioelectronics

## Abstract

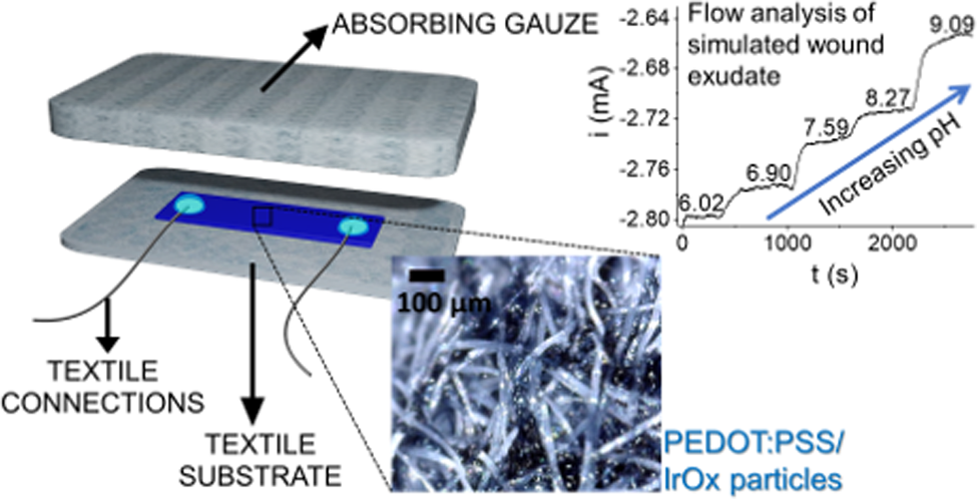

The rapid evolution of wearable technologies is giving rise to a strong push for textile chemical sensors design targeting the real-time collection of vital parameters for improved healthcare. Among the most promising applications, monitoring of nonhealing wounds is a scarcely explored medical field that still lacks quantitative tools for the management of the healing process. In this work, a smart bandage is developed for the real-time monitoring of wound pH, which has been reported to correlate with the healing stages, thus potentially giving direct access to the wound status without disturbing the wound bed. The fully textile device is realized by integrating a sensing layer, including the two-terminal pH sensor made of a semiconducting polymer and iridium oxide particles, and an absorbent layer ensuring the delivery of a continuous wound exudate flow across the sensor area. The two-terminal sensor exhibits a reversible response with a sensitivity of (59 ± 4) μA pH^–1^ in the medically relevant pH range for wound monitoring (pH 6–9), and its performance is not substantially affected either by the presence of the most common chemical interferents or by temperature gradients from 22 to 40 °C. Thanks to the robust sensing mechanism based on potentiometric transduction and the simple device geometry, the fully assembled smart bandage was successfully validated in flow analysis using synthetic wound exudate.

The rising Internet of Medical Things (IoMT) is bringing wearable devices and enabling technologies to revolutionize healthcare with a strongly patient-centric vision, inspiring innovative design and management of clinical trials. One of the medical fields that could mostly benefit from the IoMT approach is the management of nonhealing wounds.

The wound healing process is a complex cascade of physiological events, which are vulnerable to both our body status and external factors and whose dysregulation leads to impaired healing or chronicity.^1−4^ Chronic wounds typically exceeding a 3-month healing process are recognized as a major source of mortality in bed-ridden and diabetic patients and imply high treatment costs.^4−6^ Current treatments of chronic wounds rely on wound dressings tailored to the specific healing case, based on healing stage, inflammatory status, moisture level, and exuding rate. However, the wound status is typically evaluated by visual inspection and dressing change is often done unnecessarily, with persistent risk of causing a second injury or disturb the wound healing process.^4,7^ Moreover, the clinical assessment of the healing process remains challenging due to its dynamism and complexity,^8^ as well as the lack of firmly established and quantitative tools for wound monitoring over time. For these reasons, the design of novel IoMT sensors and smart dressings able to noninvasively monitor the wound site could have a dramatic impact on the outcomes of wound management and healthcare costs.

Real-time monitoring of the wound bed pH is a promising candidate for remote wound healing management. In fact, the wound pH varies according to the wound healing stages^9^ and, for this reason, noninvasive wound pH assessment can be useful to determine the wound state and the efficacy of the therapeutic strategy. While a slightly acidic pH provides optimum healing conditions to control collagen formation, increase fibroblasts activity, and hamper bacteria proliferation, more alkaline pH values (7–9) are typical of hard-to-heal wounds.^10−13^ Although pH sensing is a consolidated analytical practice and a variety of potentiometric probes are available in the market, commercial devices might be hardly adapted to wound healing monitoring. On the one hand, major constraints related to the development of wearable devices working in contact with human skin are safety and conformability, which become even more stringent when the sampling site is a lesion with impaired healing. In fact, the device should neither cause pain or discomfort nor interfere with the healing progression or contaminate the injured tissues. On the other hand, technical challenges regard the small sample volumes and its complex composition, as well as the need to evaluate spatial pH distributions rather than performing a set of time-consuming punctual measurements at specific locations.^14^ Furthermore, robustness and stability of the calibration are essential to enable real-time monitoring over time and avoid unnecessary dressing changes for sensor maintenance. Alternative to the fragile and bulky glass membrane electrodes, solid-state pH sensors based on transition-metal oxides (MOx) have experienced rapid development thanks to their unique electrical and electrochemical properties, inherent stability to harsh experimental conditions, and biocompatibility.^15,16^ Among them, IrOx finds application in several research fields, including electrocatalysis for water oxidation^17,18^ and neural probes engineering,^19−21^ and it is recognized as one of the most promising materials for pH sensing with superior sensitivity, wide detection range, and long-term stability.^22−25^ Electrochemical methods, sputtering deposition, and sol–gel process are only a few examples among the variety of preparative routes leading to IrOx films with different stoichiometries that strongly affect the final electrochemical properties.^26^ Overall, the redox equilibrium involving Ir^III^/Ir^IV^ species and H^+^ ions is the generally accepted mechanism governing the potentiometric response of the metal oxide anhydrous and hydrous forms, the latter comprising oxyhydroxides that are typically obtained after electrochemical growth and regarded as responsible for the often reported super-Nernstian sensitivity.^15,27,28^ IrOx films have been utilized for the in vitro monitoring of cells and tissues pH,^28−32^ the fabrication of transistor-based pH sensors^33,34^ and, more recently, the development of wearable pH sensors for sweat monitoring on plastic substrates^35−38^ and conductive fabrics.^39^ All in all, IrOx-based textile pH sensors have not entered the field of wound management yet.

Instead, the conjugated polymer polyaniline (PANi) is acknowledged as the gold standard in the realization of textile, solid-state potentiometric probes for quantitative pH detection. Following the pioneering work of Guinovart et al.,^40^ PANi-based, wearable pH sensors have been developed using cotton^41−43^ and polyester threads.^44^ However, despite the remarkable achievements in the design and manufacturing of textile potentiometric sensors, a major limiting factor concerns the fabrication of reliable reference electrodes by coating and printing methods, which is still at a primal level.^16,45,46^ To address this issue, our group has recently reported on a novel material-based approach for the development of chemical sensors leading to the advantages of the potentiometric transduction using a referenceless two-terminal configuration. In particular, the conductivity of a charge transport layer based on the semiconducting polymer poly(3,4-ethylenedioxythiophene) (PEDOT) doped with poly(styrene sulfonate) (PSS) is reversibly modulated by spontaneous electrochemical reactions occurring between the analyte and a potentiometric transducer placed in intimate contact with the semiconductor. Such a sensor architecture relies on a mechanism called electrochemical gating and is easily adapted to unconventional substrates. In fact, textile Cl^–^ and pH two-terminal sensors were realized on natural and synthetic fibers^47,48^ as well as bioceramic fabrics^49^ for wearable applications. This report describes the development of a novel two-terminal sensor for pH monitoring that is based on chemically synthesized IrOx particles (IrOx Ps) embedded in a PEDOT:PSS thin film. We demonstrate that the conductivity of the here proposed novel hybrid material reversibly changes with pH variations due to spontaneous redox reactions involving the metal oxide particles, leading to remarkable sensing performances in terms of reproducibility, stability, and accuracy. Thanks to the robustness of the potentiometric transduction and the simple two-terminal geometry, the PEDOT:PSS/IrOx Ps sensor was encased in a wound dressing structure targeting real-time pH monitoring. To the best of our knowledge, the here reported smart bandage is the first example of textile pH sensor validated for flow analysis using synthetic wound exudate, thus paving the way for next-generation IoMT devices for wound management.

## Experimental Section

### Chemicals and Buffers

CLEVIOS PH1000 suspension (PEDOT:PSS) was purchased from Heraeus. (3-Glycidyloxypropyl)trimethoxysilane (GOPS), sodium dodecylbenzenesulfonate, potassium nitrate, silver nitrate, potassium hydroxide, sodium hydroxide, sodium chloride, nitric acid, acetic acid, 85% phosphoric acid, and boric acid were purchased from Sigma-Aldrich. Potassium chloride was bought from Fluka. Ethylene glycol (EG) was obtained from Carlo Erba. IrCl_4_ was purchased from Alfa Aesar. Silicone elastomer and curing agent for the preparation of PDMS were obtained from Sylgard. The conductive silver paste was obtained from RS Components. All chemicals were of reagent grade or higher. The ionic strength of all solutions was buffered with 0.1 M KNO_3_. The phosphate buffer solution (PBS) was made from 0.1 M KH_2_PO_4_ and corrected to pH 7.00 by adding 1 M KOH. The universal buffer (U. B.) solution was made from 0.01 M H_3_PO_4_, 0.01 M H_3_BO_3_, and 0.01 M CH_3_COOH in 0.1 M KNO_3_. Simulated wound exudate (SWE) was prepared by mixing 0.142 M NaCl and 0.0025 M CaCl_2_·2H_2_O while using 0.025 M TRIS and 0.005 M Histidine·HCl·H_2_O as a pH buffer.

### Apparatus

All potential-controlled and open-circuit potential (OCP) measurements were carried out in a single compartment three-electrode cell, using a potentiostat (CH Instrument 660 C). Electrode potentials were measured with respect to an aqueous saturated calomel electrode (SCE) and a Pt gauze was used as the counter electrode (CE). pH sensing measurements with the two-terminal sensor were carried out with a Source-measure Unit (Keysight B2902A). A combined glass electrode (Amel 411/CGG/12) connected to a pH meter (Amel instruments 338) was employed for pH measurements. Analyses combining atomic force microscopy (AFM) with Kelvin probe force microscopy (KPFM) were performed with a Park NX10 system operated in noncontact mode using point probe plus (PPP)-Noncontact/soft tapping mode (NCST) Au probes (nanosensors). In the KPFM mode, an AC signal of 1 V was applied to the tip at 17 kHz. Measurements were carried out under controlled ambient conditions. The same tip was used for all measurements. Tip wear effects could be excluded here due to strict noncontact operation minimizing mechanical shear forces between sample and tip. To evaluate the absolute scale of work function, a surface potential of pure Au was measured and used as reference value. UV-Vis measurements were performed using a Hewlett-Packard 8453 diode array spectrophotometer. The IrOx Ps suspension was diluted in distilled water (dilution factor 2.5) and four samples at different pH were prepared upon addition of 1% NaOH. The samples were tested in a quartz cuvette in the wavelength range 200–800 nm. For the DLS analyses, IrOx Ps suspension was tested as such using a Zetasizer Nano, Malvern Panalytical. An HPLC pump (LabFlow 1000) was employed for the flow analyses.

### Synthesis of IrOx Particles

IrOx Ps were chemically synthesized starting from a 2 mM IrCl_4_ aqueous solution and adapting an already reported procedure.^50^ NaOH (10 wt %) was added dropwise to the brown solution under stirring until the pH turned basic, giving a light yellow color. The solution was then heated at 90 °C for 1 h under stirring, until a homogeneous light blue color appeared. Therefore, it was rapidly cooled down in an ice bath, and 3 M HNO_3_ was added dropwise until the solution pH turned acidic. Finally, the solution was kept under stirring for 80 min at room temperature. At the end of this procedure, the dark-blue suspension containing IrOx Ps was aged at 4 °C, protected from light sources, for 24 h. The solution was stored for a maximum period of 2 months at 4 °C, and it was brought to room temperature prior to use.

### Fabrication of Sensors on Glass

The device is composed of two parallel Cr/Au electrodes and a PEDOT:PSS stripe between them. The Cr/Au (10/40 nm) tracks were deposited on a glass slide via thermal evaporation using a physical mask. CLEVIOS PH1000 suspension was mixed with EG, dodecylbenzene sulfonic acid, and GOPS in the following volumetric ratio 93.75:5:0.25:1. The solution was sonicated for 10 min before spinning and filtered using 1.2 μm cellulose acetate filters (Sartorius) before the deposition. The substrates were cleaned in sequential sonicating baths of deionized water, acetone, and isopropanol for 15 min. Then, the substrates were masked and the PEDOT:PSS solution was spin-cast at 500 rpm, for 3 s with an acceleration of 500 rpm/s. Afterward, the devices were thermally annealed for 1 h at 140 °C. The IrOx Ps suspension as such was employed as an electrolyte solution to carry out the electrodeposition. A standard electrochemical cell was used where the polymer film of a two-terminal device was the WE and a Pt wire and an SCE were the CE and RE, respectively. The potential of the WE was scanned between 0 < *E* < 1 V vs SCE for 100 cycles at 100 mV s^–1^. The sensor was then thoroughly rinsed with distilled water and stored under ambient conditions.

### Fabrication of Textile Sensors

A total of 25 smart bandages for pH sensing were fabricated using the following procedure. A conducting ink made of 78% v/v PH1000, 20% v/v EG, and 2% v/v GOPS was warmed up in an oven at 60 °C to lose about 40% of the initial weight to obtain the suitable viscosity for the deposition. Then, a mask was employed to screen-print the desired sensor pattern (2 × 0.5 cm^2^ stripe) onto the sterile dressing applying 3 mL of the conductive ink over the mask and performing four streaks with the aid of a metal spatula to force the ink through the mask shape. Subsequently, the device was put on a hotplate at 150 °C for 10 min to anneal the ink, allowing the partial reticulation of PEDOT:PSS chains by GOPS. After cooling, two commercial conductive threads were sewn at the edges of the sensor area and a small amount of conductive silver paste was applied to reduce the contact resistance between the conductive polymer and the conducting threads. Then, the textile devices were heated to 150 °C on a hotplate and a mixture of PDMS-curing agent (9:1 w/w) was applied over the silver paste to obtain electrical insulation. The IrOx Ps were electrochemically deposited on the textile substrate following the same procedure described for the sensors made on glass. After that, the smart pH sensing bandage was assembled in a sandwich-like manner with an absorbing layer.

### pH Measurements

The two-terminal sensors were connected to the source-measure unit to perform pH detection. One terminal was connected to ground and a fixed potential of −200 mV was applied to the other, while the generated current was measured vs time. All tests were performed in U. B., and the solution pH was changed by dropwise addition of 1 M KOH or 1 M HNO_3_ under stirring. The exact pH value of the solution, following each addition, was measured in blank experiments using the glass electrode. Note that the number of independent repetitions carried out for repeatability tests is denoted by N. Five textile sensors were tested in flow analysis using U. B. or SWE solutions buffered at different pH with TRIS and Histidine·HCl·H_2_O, whose pH was adjusted through the addition of known volumes of 1 M KOH and HCl using a glass electrode.

## Results and Discussion

### Preparation of the PEDOT:PSS/IrOx Ps Film

**Figure 1:**
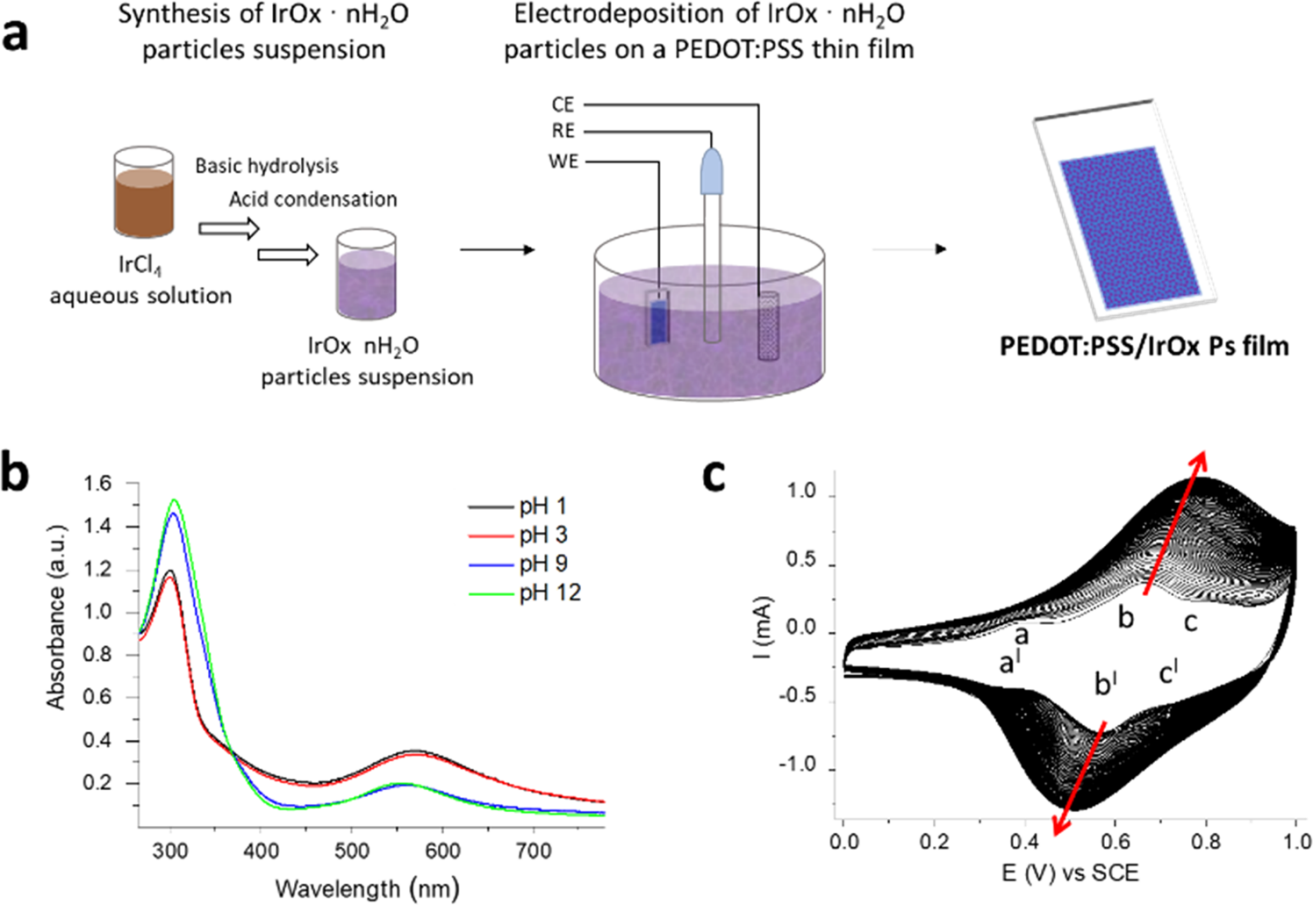
Synthesis of the PEDOT:PSS/IrOx Ps film. (a) Preparation of the composite film on a glass substrate, including IrOx particles synthesis and electrodeposition on a PEDOT:PSS film. (b) UV–vis characterization of the IrOx Ps suspension at different pH values. Dilution factor = 2.5. (c) Electrochemical deposition of IrOx Ps on the PEDOT:PSS film. Scan rate = 100 mV s^–1^.

The first report concerning the synthesis of a stable, blue colloidal suspension of IrOx·*n*H_2_O nanoparticles dates back to 1908 and is based on the thermally assisted basic hydrolysis of the Ir^IV^ complex [IrCl_6_]^2–^.^51^ Since then, syntheses of both capped^52,53^ and ligand-free^50,54−56^ IrOx·*n*H_2_O NPs have been reported and eventually combined with anodic deposition,^50,52,54,55,57,58^ spin coating,^56^ self-assembly,^53^ and ink-jet printing^59^ techniques to obtain functional electrochemical interfaces for water splitting and pH sensing. In this study, the pH-sensitive semiconductor PEDOT:PSS/IrOx Ps was prepared using a double-step procedure consisting of (i) IrOx·*n*H_2_O Ps synthesis and (ii) electrodeposition on a PEDOT:PSS film (Figure 1a). A deep-blue aqueous suspension of IrOx·*n*H_2_O Ps was obtained via basic hydrolysis and subsequent acidification of a 2 mM solution of Ir^IV^, and the ultraviolet–visible (UV–vis) characterization of the as-synthesized sample is reported in Figure 1b. It has been shown that the introduction of the additional acidic condensation step following the hexachloroiridate complex hydrolysis promotes the protonation of [Ir(OH)_6_]^2–^ and the formation of Ir^IV^–O–Ir^IV^ linkages, leading to a quantitative conversion to stable, ligand-free IrOx·*n*H_2_O that has been thoroughly investigated by UV–vis spectroscopy.^50^ Despite the here chosen, cheaper Ir^IV^ salt as the starting material, i.e., IrCl_4_, the results are in agreement with the literature. An isosbestic point around 370 nm highlights the coexistence of two species, i.e., [Ir(OH)_6_]^2–^ and IrOx·*n*H_2_O colloid, which absorb at 304 and 574 nm, respectively, and are in equilibrium depending on the solution pH. As expected, the absorption band at 574 nm increases in intensity upon acidification (Figure 1b), suggesting the promoted formation of IrOx·*n*H_2_O colloids. The distribution profile of the ligand-free IrOx·*n*H_2_O Ps in the as-synthesized acidic aqueous suspension was obtained by dynamic light scattering (DLS) and is reported in Figure S1. The particles have a mean diameter of (1.1 ± 0.5) μm and a *Z* potential of (−18 ± 1) mV. The IrOx·*n*H_2_O Ps suspension was directly used as the electrolytic solution to carry out the electrochemical deposition of the particles onto a PEDOT:PSS film. Figure 1c shows the voltammogram recorded upon application of 100 potentiodynamic cycles in the range 0 < *E* < 1 V vs SCE to the PEDOT:PSS working electrode. The current increase suggests that a growing number of particles are embedded upon cycling into the semiconductor film. Three faradic peaks, labeled as a-a^I^, b-b^I^, and c-c^I^, can be identified and, to interpret the shape of such a voltammogram, the strongly acidic pH of the electrolyte solution must be taken into account. The peaks b-b^I^ and c-c^I^ are ascribable to the Ir^III^/Ir^IV^ and Ir^IV^/Ir^V^ redox couples, respectively.^60,61^ Differently, the peaks a-a^I^ should be associated with the altered electrostatic interaction between PEDOT and its sulfonate counterion due to the pH of the electrodeposition solution. This hypothesis was verified by cycling the bare PEDOT:PSS film in pH buffers below and above the pKa of PSS (e.g., 1.50),^62^ showing that a faradic feature appeared when the counterion is protonated (Figure S2).

### Morphological Characterization

**Figure 2:**
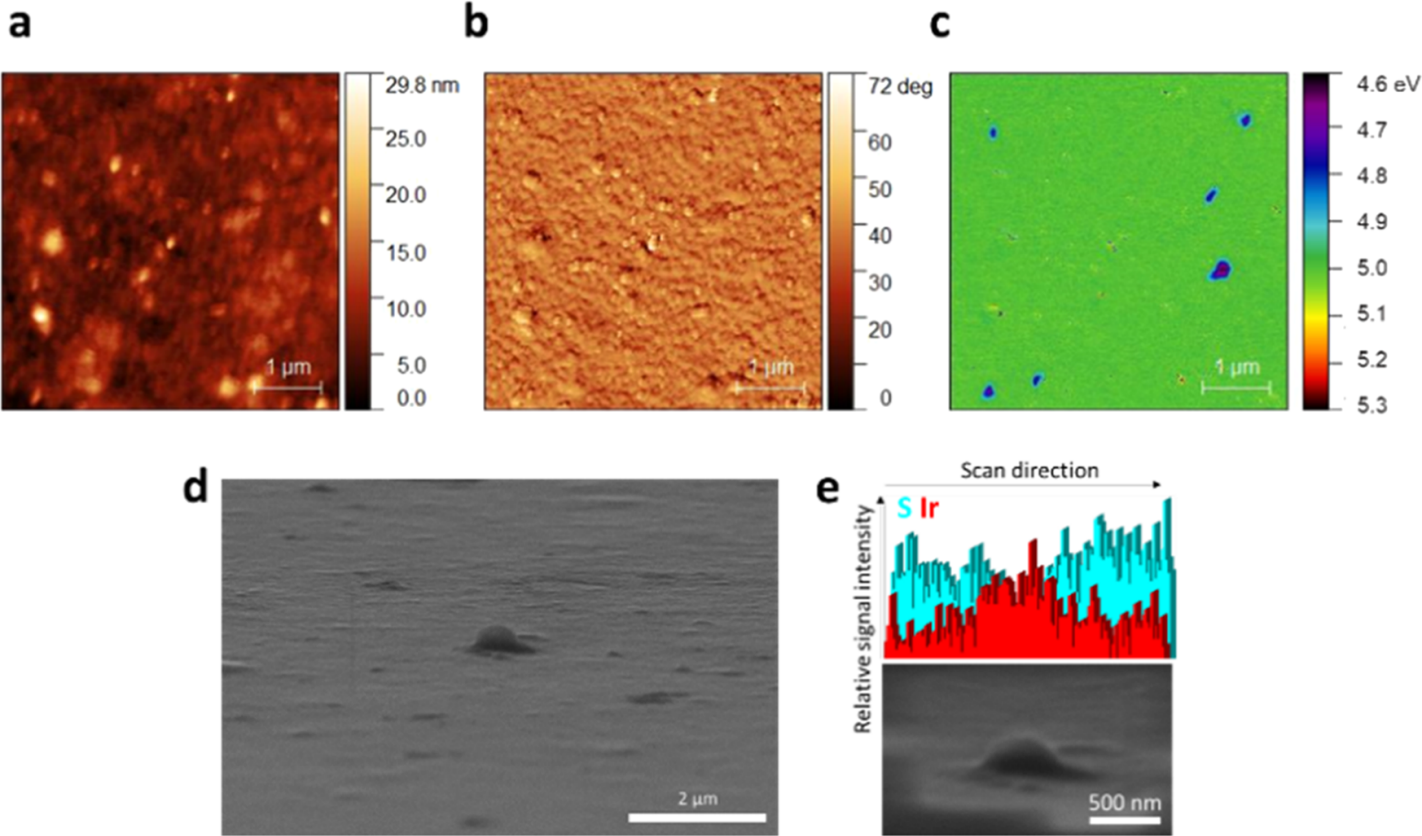
Morphology of the PEDOT:PSS/IrOx Ps film. (a) AFM height, (b) phase images, and (c) KPFM map of the composite film. (d) SEM imaging of the tilted sample surface and (e) EDS profile of the portion localized around a protruding particle.

The morphology of the resulting PEDOT:PSS/IrOx Ps film was investigated by AFM (Figure 2a–c). Different from the flattened surface that is typically observed for spin-coated PEDOT:PSS films,^49,63^ discrete globular structures are visible in the height profile (Figure 2a), being consistent with the RMS roughness increase from (0.6 ± 0.1) nm for the pristine PEDOT:PSS film to (1.0 ± 0.1) nm following electrodeposition of the particles. Differently, the thickness of PEDOT:PSS before and after inclusion of IrOx Ps showed no statistically relevant changes, from (6.1 ± 0.9) × 10^2^ nm to (6.3 ± 0.9) × 10^2^ nm. The presence of particles of about 300 nm diameter can be noticed by observing the KPFM map (Figure 2c). Emerging from the PEDOT:PSS background of 5 eV,^47^ a work function (WF) of 4.7/4.8 eV was calculated from the KPFM potential for IrOx Ps, which is consistent with the WF ranges previously reported for crystalline and amorphous iridium oxides.^64,65^ A sample area showing a protruding particle was also analyzed by SEM-EDS (Figure 2d,e). By tilting the sample surface, an EDS map of the site including the particle was acquired obtaining a background signal due to the S atoms in the polymer backbone and a peak for Ir in correspondence of the particle position. Based on these results, we hypothesize that the particles are embedded within the PEDOT:PSS film, rather than confined on the polymer surface, which is consistent with the three-dimensional swelling of PEDOT films in aqueous environment during electrochemical deposition.

Indeed, it is worth noting that these particles are not clearly visible from the AFM height profile and phase map reported in Figure 2a,b and are only detectable by electrically biasing the AFM tip (Figure 2c). To investigate the effect of the substrate, the electrodeposition was carried out on a thin film of evaporated Au using the same experimental conditions. Different from the three-dimensional PEDOT:PSS structure, Au cannot swell in aqueous environment and the AFM-KPFM characterization reported in Figure S3 shows good matching between the sample topography and KPFM profile. Smaller IrOx particles of around 100 nm diameter decorate the gold surface that, however, reduces the KPFM resolution due to the use of a Au AFM probe.

### Electrochemical Characterization of the PEDOT:PSS/IrOx Ps Film

**Figure 3:**
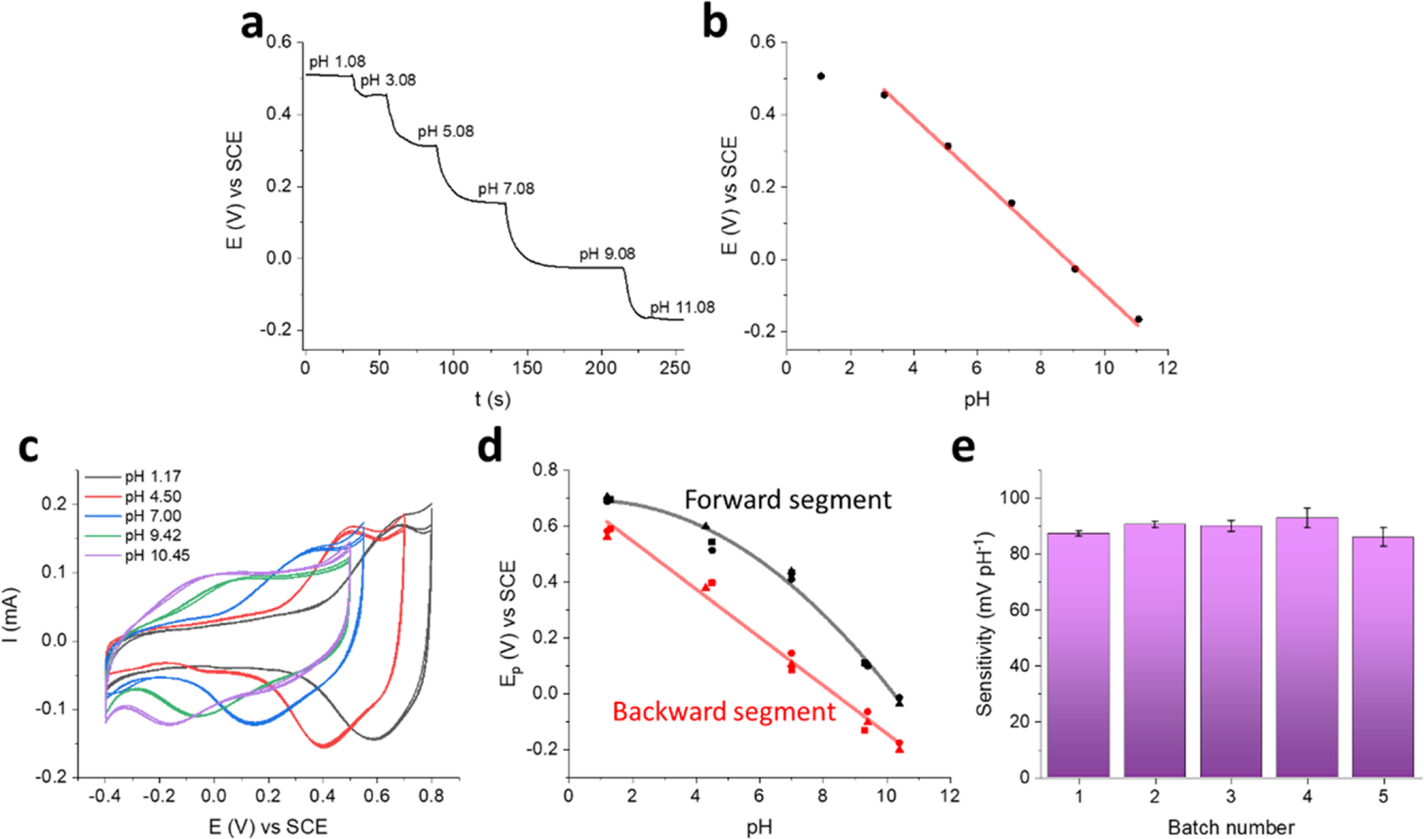
Electrochemical characterization of the PEDOT:PSS/IrOx Ps film. (a, b) Open-circuit potential response of a PEDOT:PSS/IrOx Ps film during 1 M KOH additions in U. B. and corresponding calibration plot. *R*^2^ = 0.998. (c) Cyclic voltammograms of the same film in buffers at different pH values. Scan rate = 20 mV s^–1^. (d) Peak potentials vs pH plots of the forward (black) and backward (red) segments from CV characterizations of three different PEDOT:PSS films after functionalization with IrOx Ps coming from the same synthesis batch. (e) Reproducibility of the backward peak potential sensitivity among different IrOx Ps synthesis batches.

The pH transducing ability of Ir oxides has been thoroughly documented in the literature and has long been exploited to build potentiometric pH sensors.^22−25^ The electrochemical behavior of the PEDOT:PSS/IrOx Ps film was investigated in response to pH variations of the electrolyte solution and is shown in Figure 3. Potentiometric measurements were carried out by recording the zero-current electrochemical potential (open-circuit potential, OCP) of the film during base additions to a Universal Buffer solution (U. B.). The E vs pH plot shows a linear relationship in the pH range 3–11 (*R*^2^ = 0.998) with a slope of (−81 ± 2) mV pH^–1^. It is worth noting that the functionalization with IrOx Ps is essential to provide the organic semiconductor with pH sensing properties (see Figure S4). The electrochemical response of PEDOT:PSS/IrOx Ps was also studied by cyclic voltammetry (CV) using different pH buffers with the same ionic strength as electrolyte solutions (Figure 3c). The voltammograms are characterized by a couple of quasi-reversible redox peaks, whose peak potential (*E*_p_) shifts toward more cathodic values as the pH increases. The peak position at acidic pH (black line) suggests that it corresponds to the main redox wave observed during the potentiodynamic deposition of the particles (Figure 1c), i.e., the system b-b^I^. This indicates that the Ir^III^/Ir^IV^ redox couple is responsible for pH transduction, possibly according to one of the following reactions^15,66^
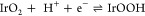
1
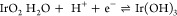
2which can be described by the Nernst equation
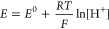
where *E*^0^ is the standard reduction potential of the redox couple, *R* is the gas constant, *T* is the absolute temperature, and *F* is the Faraday constant, thus explaining the OCP decrease in Figure 3a and the shift of the peak potential toward more cathodic values in Figure 3c as pH increases.

The values of E_p_ for the forward and backward segments can be plotted vs pH (Figure 3d), showing nonlinear and linear correlations, respectively. In this case, a backward slope of (−86 ± 3) mV pH^–1^ was calculated in the pH range 1–10 (*R*^2^ 0.982). This parameter was chosen to compare the PEDOT:PSS/IrOx Ps films prepared from five different IrOx Ps synthesis batches (Figure 3e). With a mean slope of (−89 ± 3) mV pH^–1^ and a relative standard deviation (%RSD) equal to 3%, it can be concluded that the overall procedure for the preparation of PEDOT:PSS/IrOx Ps films shows very good reproducibility.

### pH Sensing Performance of the Two-Terminal Sensor

**Figure 4:**
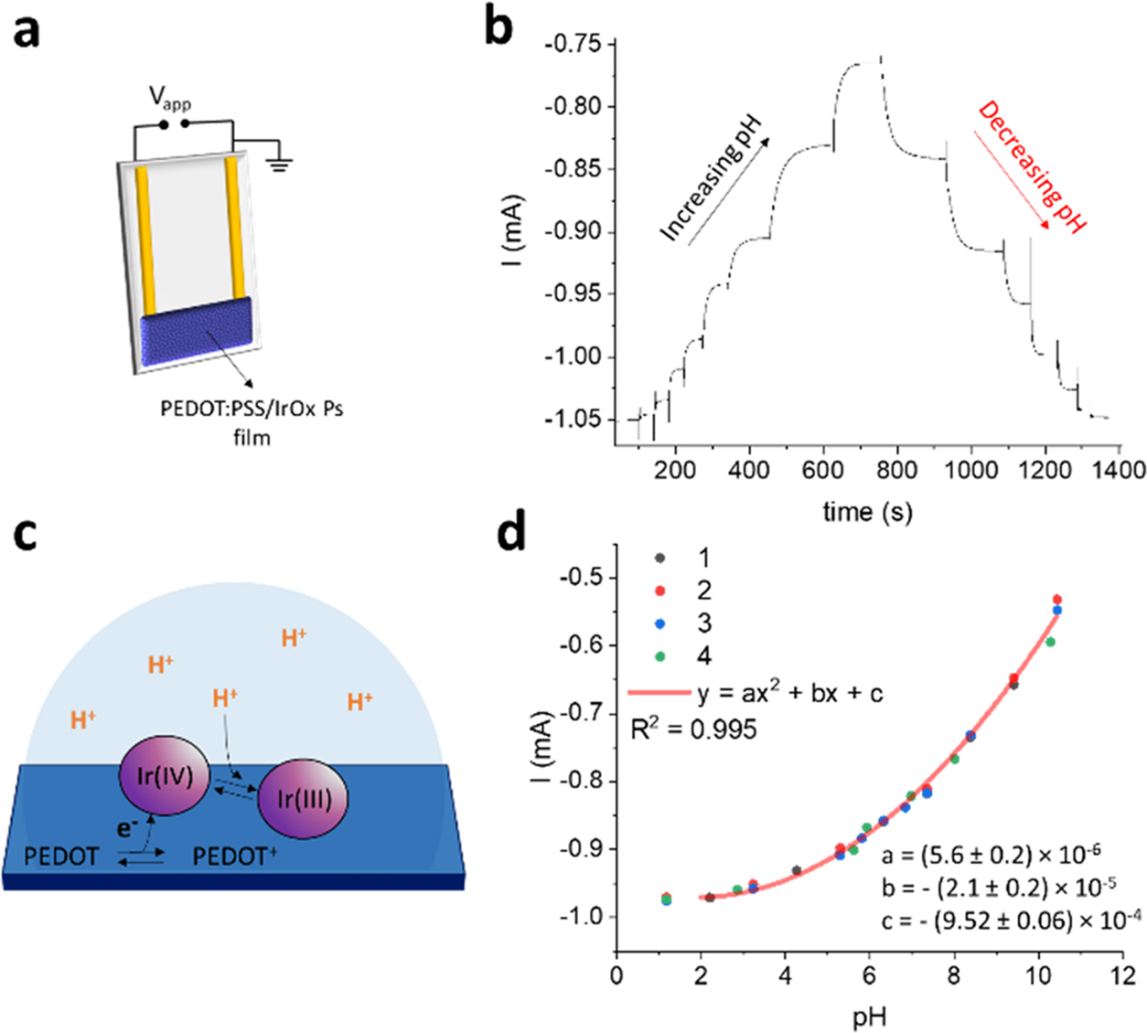
Response of the two-terminal PEDOT:PSS/IrOx Ps pH sensor. (a) Scheme of the two-terminal sensor. (b) Sensor response recorded in U. B. during 1 M KOH and 1 M HNO_3_ additions. *V*_app_ = −200 mV. (c) Proposed sensing mechanism in the two-terminal pH sensor. (d) Polynomial curve describing the sensor response obtained from the four independent *I*/*t* measurements reported in Figure S6 (*N* = 4).

The electrochemical characterization highlights that not only the material developed here is a pH transducer, but also it changes its redox state in response to pH variations spontaneously, thus being a suitable platform for the development of electrochemical pH sensors. In general, a solid-state configuration avoiding brittle components and complex readout electronics is desired in view of wearable applications. For this reason, the PEDOT:PSS/IrOx Ps film was patterned between two gold electrodes, leading to the two-terminal architecture depicted in Figure 4a. A small potential difference (*V*_app_) is applied between the two electrodes, and the generated current flowing across the semiconducting film is measured versus time while the two-terminal device is immersed in 0.1 M KNO_3_ U. B. As the pH of U. B. is varied upon controlled additions of base or acid, stepwise decrements or increments of the recorded current are observed, respectively (Figure 4b). *V*_app_ was chosen based on the readout signal showing the best signal-to-noise ratio and reversibility to pH variations (Figure S5), the optimized value being −200 mV. A scheme of the proposed sensing mechanism is reported in Figure 4c. An acidic environment promotes the reduction of Ir^IV^ sites to Ir^III^, causing the concomitant extraction of electrons from the PEDOT:PSS film. Therefore, the semiconductor is oxidized leading to an increase of charge carriers concentration (PEDOT^+^) and of the current flowing across the PEDOT:PSS film. Conversely, a current decrease is observed upon base additions, as the charge transfer reaction between Ir species and PEDOT:PSS causes a depletion of holes in the semiconductor and reduces its conductivity.

The peculiarity of the two-terminal sensor is that, if the steady-state current is plotted vs pH (Figure 4d), a nonlinear response in the pH range 2–11 is obtained, which can be interpolated by a second-order polynomial curve with the equation
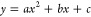
In analogy with a parabolic function in geometry, the coefficients *a*, *b*, and *c* are linked to the concavity/steepness, position of the axis of symmetry, and y-intercept of the curve, respectively. Although a similar response might look unusual, the robustness of the quadratic regression was demonstrated during repeatability measurements with randomized additions of acid and base to U. B. (Figure S6), following the protocol suggested in other literature reports.^67,68^ The response time of sensor (*t*_90_) varies according to the pH range and is 7, 19, and 70 s in the acidic, intermediate, and basic pH intervals, respectively. The pH-dependent response time is likely due to the different interactions of H^+^ and OH^–^ with metal oxides and the faster diffusion speed of H^+^ species, which are dominant in acidic solutions.^16,69,70^ The reproducibility was investigated by repeatedly testing (*N* > 3) three different devices, and Figure S7 reports the calibration plots. The current response highlights a 5% variability in the baseline current that is ascribed to geometrical differences, while the normalized current response (NCR) plots eliminate the geometry contribution and facilitate the comparison among different devices. In this case, the resulting values of the coefficients a, b, and c in the nonlinear regression were (5.7 ± 0.5) × 10^–3^, (2.3 ± 0.6) × 10^–2^, and (3 ± 1) × 10^–2^, respectively.

If the sensor response is analyzed in smaller pH intervals, two segments of the polynomial curve are identified that can be reliably interpolated with a straight line. It is in fact possible to describe the nonlinear sensor behavior using linear responses within limited pH ranges, thus leading to practical advantages in the expression of the analytical performances, such as pH sensitivity. We report the calibration plots in the pH ranges 2.5–5.2 and 6.4–9.3 (Figure S8) pointing out the linear *I*/pH correlation, for which sensitivities of (20 ± 1) μA pH^–1^ and (59 ± 4) μA pH^–1^ were calculated, respectively. Interestingly, the highest sensitivity is obtained in the pH interval matching the range of interest for wound healing monitoring.

The effect of film composition on the pH sensing performance of the two-terminal device was also investigated (Figure S9). The relative amount of IrOx Ps within the semiconductor film was varied by changing the number of deposition cycles during the electrochemical functionalization. In particular, 10, 50, 100, and 200 deposition cycles were performed and the resulting films were analyzed by SEM-EDS to estimate the Ir/S atomic ratio. The four PEDOT:PSS/IrOx Ps two-terminal devices were therefore tested for pH detection in U. B. As evident from Figure S9c, not only the relative amount of Ir atoms increases with the number of deposition cycles but also it well correlates with the NCR sensitivity values in the basic pH range. This suggests that further inclusion of IrOx Ps within the polymer film would improve the sensing performance in the pH range of interest. Nonetheless, the relative gain in pH sensitivity is penalized by a longer preparation time and thus 100 cycles have been employed for the fabrication of sensors.

**Figure 5:**
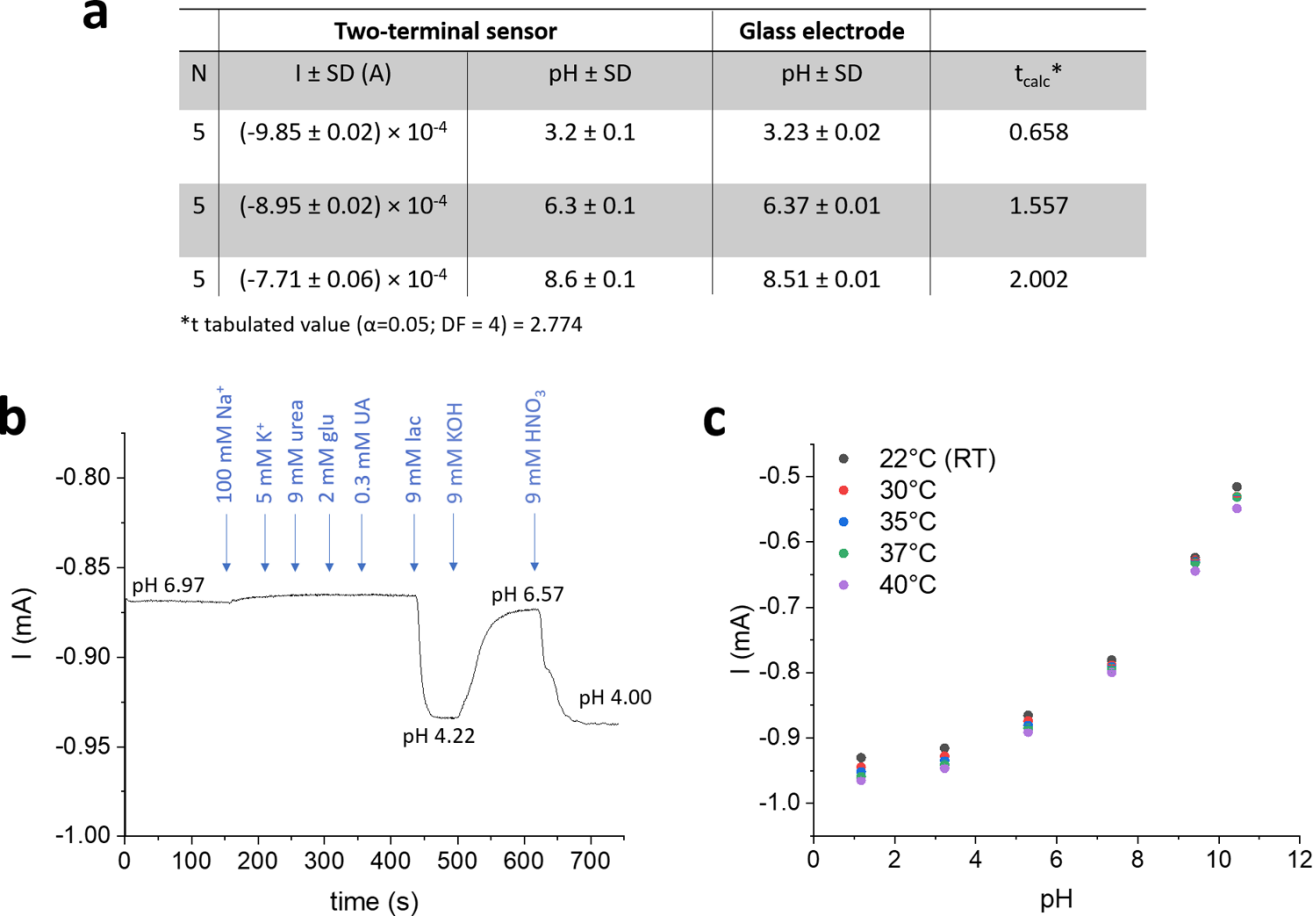
Validation of the two-terminal PEDOT:PSS/IrOx Ps pH sensor. (a) Comparison between a precalibrated two-terminal sensor and a glass membrane electrode for pH determination in U. B and statistical analysis. (b) Interference study in U. B. adjusted to pH 7.00 by adding controlled amounts of chemical species that are typically found in wound exudate (Na^+^, K^+^, glucose, uric acid, urea, and lactate). (c) Effect of temperature gradients on the sensor response.

With the aim to assess the applicability of the PEDOT:PSS/IrOx Ps two-terminal device in real-life applications, the sensor response was validated using a consolidated electroanalytical method for pH measurement under laboratory conditions. We recorded the current generated after immersing a precalibrated device in 3 U. B. aliquots with random pH and calculated the experimental pH value from the calibration curve, which was compared with the one measured by a glass electrode using a two-tailed Student’s *t* test. The results are summarized in Figure 5a, and a good agreement was found between the two methods (α = 0.05). Focusing on the target wound healing monitoring, the effects of interfering species and temperature were evaluated. Controlled amounts of the main chemical species that are found in wound exudate, i.e., Na^+^ and K^+^ ions, urea, glucose (glu), uric acid (UA), and lactate (lac), were added at their typical concentrations^71^ to a U. B. solution at pH 7.00 (Figure 5b). While the addition of lactate causes the expected variation of the recorded current due to its acid–base properties, the addition of 0.1 M Na^+^ leads to a 0.05% current decrease that can be ascribed to the pronounced increase of the ionic strength of the solution (from 0.1 M due to KNO_3_ in the U. B. recipe to 0.2 M) and, thus, of the solution pH due to the variation of the activity coefficients.

The remaining potentially interfering species affected neither the sensor response nor its ability to detect pH variations when KOH or HNO_3_ was finally added as control. Due to its facile electrochemical oxidation at PEDOT-based interfaces, ascorbic acid typically represents the major interference-related issue in biofluids sensing. However, to the best of our knowledge, this biomolecule is not present in the wound environment^71^ and, for this reason, it was excluded from this interference study. Conversely, a high protein loading is generally found in both healing and nonhealing wound fluids, which is likely to produce some fouling effect at the sensing interface after prolonged use in real samples.

Another essential parameter to consider for the development of wearable sensors is temperature. Indeed, not only the temperature of our skin is substantially different from the standard room temperature conditions, but also the temperature of the wound environment is variable and can exceed 37 °C.^72^ The effect of temperature was analyzed by calibrating the two-terminal sensor inside a thermostatic electrochemical cell and adjusting the temperature of the U. B. solution where the device was immersed from 22 to 40 °C. The variation of the coefficient a of the polynomial curve, expressed as %RSD, within the temperature range under investigation is 0.4% °C^–1^ and lies in the uncertainty value associated with this parameter during repeatability studies (Figure 4d), thus indicating that the sensor response is not affected by temperature gradients. The sensor stability was studied during daily and long-term use. The results reported in Figure S10 demonstrate that a drift of the measured signal with a %RSD below 2% is achieved under both acidic and basic pH conditions over a 5 h experiment. Despite a baseline shift that could be corrected by normalizing the recorded current, the sensor retains its performance even after 40 days, during which it was stored in air under ambient conditions.

### Origin of the Two-Terminal Sensor Response

The sensing mechanism of the two-terminal pH sensor was investigated by focusing on the electrochemical interaction between IrOx Ps and the PEDOT:PSS film. Considering the intimate contact between the potentiometric transducer (IrOx Ps) and the charge transport layer (PEDOT:PSS), we ascribe the sensor response to the spontaneous electrochemical gating realized at the interface between the two elements.^47,49^ In fact, IrOx Ps behave as a multitude of miniaturized gate electrodes integrated within the semiconducting channel of a transistor, whose conductivity is reversibly switched on/off upon gating. In this case, however, the gate action originates from the spontaneous redox reactions occurring at the IrOx Ps/pH buffer interface that do not need an external bias to take place. The working principle is analogous to that reported in our previous work, in which a PEDOT:PSS film was functionalized with Ag/AgCl NPs to obtain a two-terminal sensor for Cl^–^.^47^ This has two major consequences: (i) a pH-dependent current modulation in the two-terminal device occurs, which can be quantitatively exploited as an analytical signal for pH sensing and whose robustness and reliability are supported by the potentiometric transduction, and (ii) the need of a physically separated gate terminal is eliminated, as the sensing process is realized through spontaneous redox reactions involving IrOx Ps that are placed in direct electrical contact with the polymer film.

**Figure 6:**
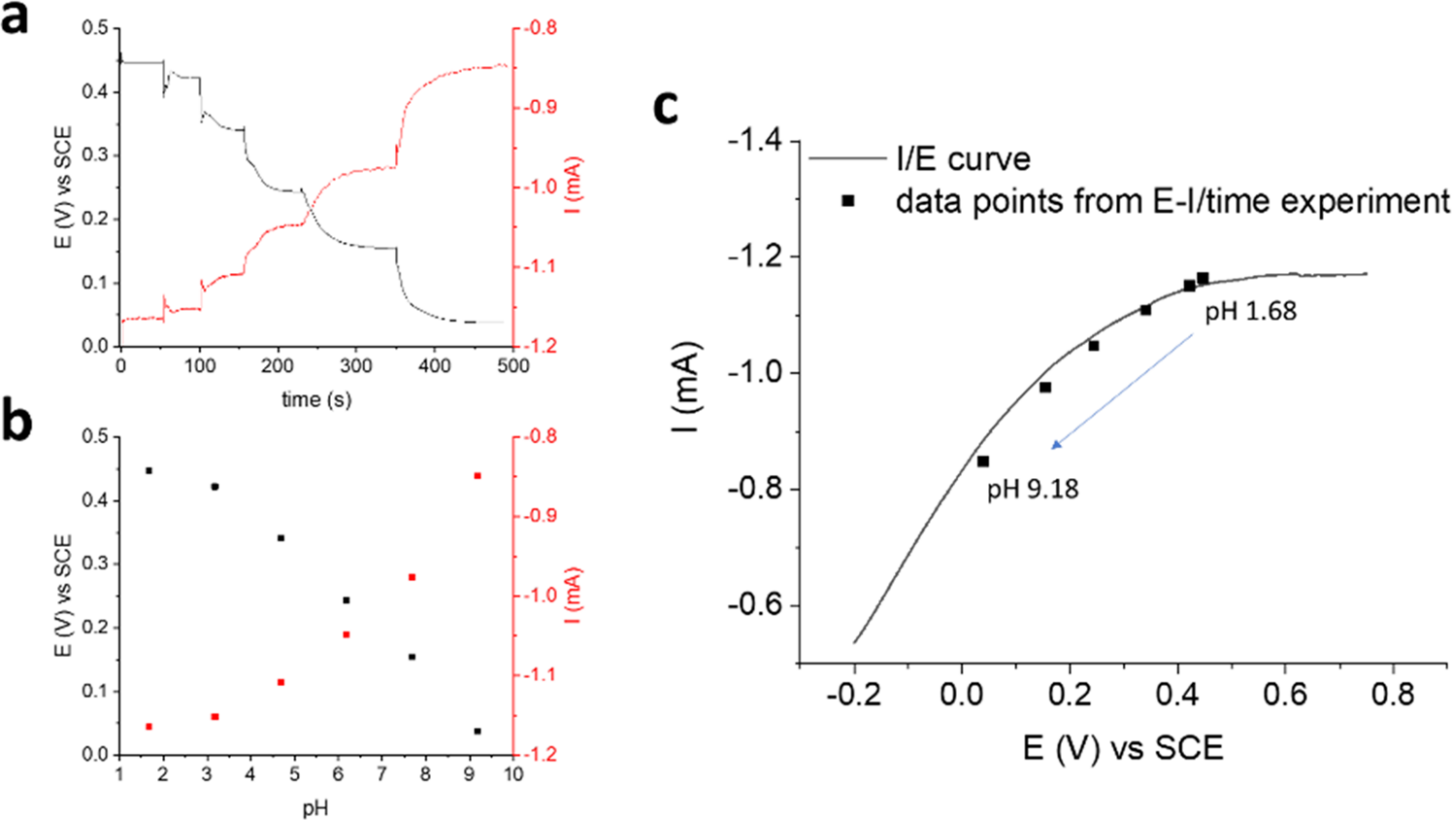
Investigation on the polynomial response of the two-terminal sensor. (a) Simultaneous measurement of the electrochemical potential of the grounded terminal and the current flowing across the semiconductor during pH detection and (b) the corresponding calibration plots. *V*_app_ = −200 mV. (c) Superimposition of the data collected from (a) and an *I*/*E* curve recorded by sweeping the electrochemical potential of the grounded terminal in U. B. Scan rate = 10 mV s^–1^.

To understand the reason for the nonlinear sensor response, the electrochemical potential of the grounded terminal was measured with respect to a reference electrode during pH detection with the two-terminal sensor (see Figure S11 for the experimental setup). Figure 6a,b shows the *E* and *I* curves recorded over time upon base additions and the corresponding calibration plots, respectively. As already mentioned, the pH-dependent current variations result from charge transfer reactions involved in the electrochemical gating mechanism, which is driven by the potentiometric pH transducer IrOx and, in fact, *E* and *I* exhibit complementary trends. Moreover, the *I* vs *E* curve of the PEDOT:PSS/IrOx Ps film was recorded by sweeping the potential of the grounded terminal between −0.2 and + 0.8 V vs SCE and revealed linear and saturation regions of conductivity, which are typical of the organic semiconductor (Figure 6c).^73^ If the data in Figure 6b are plotted in the same figure, it stands out that the sensor response is superimposed to a portion of the *I*/*E* curve that lies between the two regions. Consequently, it can be concluded that the polynomial response of the PEDOT:PSS/IrOx Ps sensor originates from the nonlinear variation of charge carrier mobility in the semiconductor within the electrochemical potential window of interest.

### Design of a Smart Dressing for pH Monitoring

**Figure 7:**
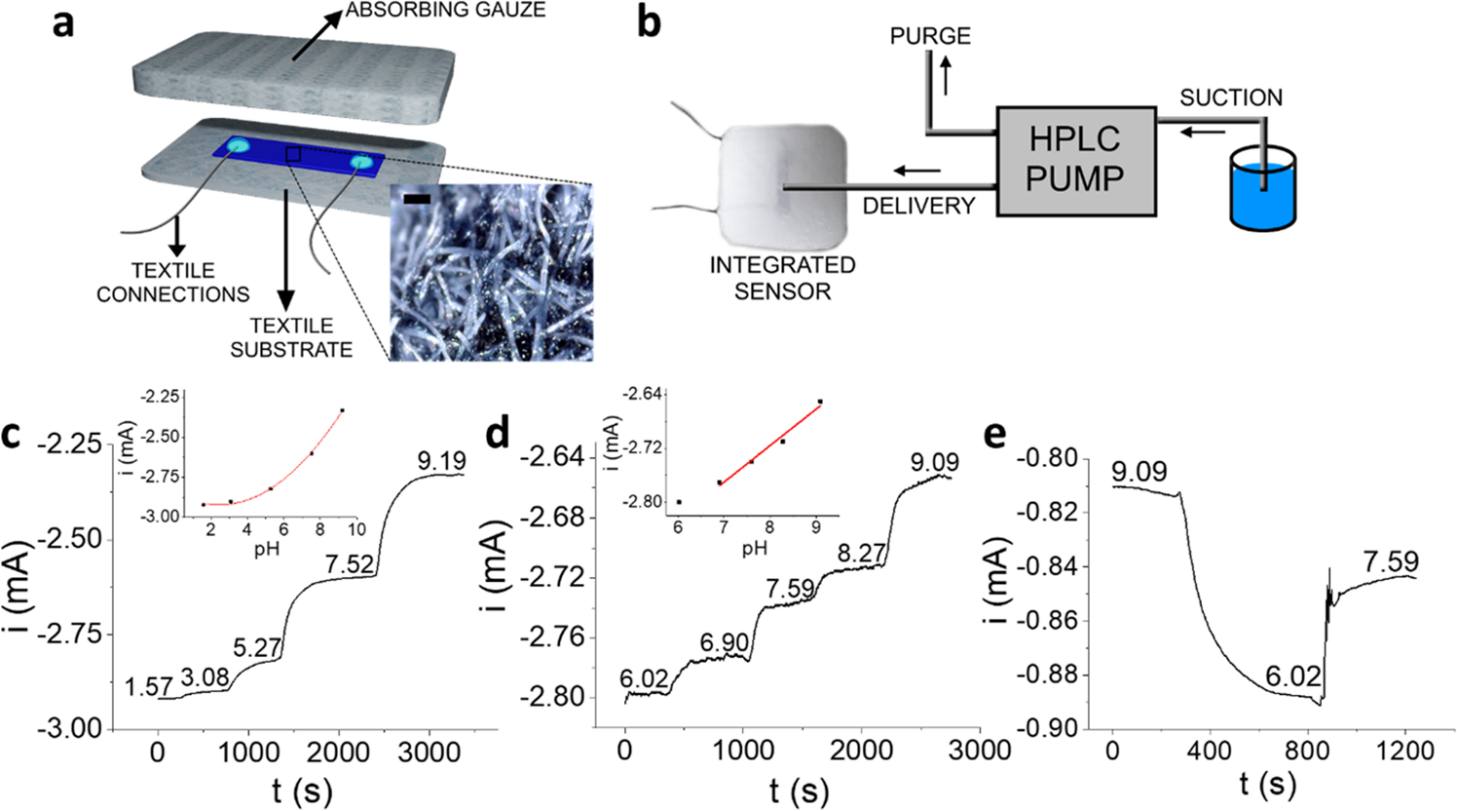
Design of the pH sensing smart dressing. (a) Scheme of the smart dressing for wound healing monitoring, comprising the PEDOT:PSS/IrOx Ps two-terminal sensor printed on a textile substrate and the absorbing gauze. Inset: picture of the textile PEDOT:PSS/IrOx Ps two-terminal sensor (scale bar = 100 μm). (b) Experimental setup used for flow experiments with the smart pad. (c) Real-time, in-flow response of the wearable sensor in U. B. at different pH values; inset: calibration plot (*R*^2^ = 0.996). (d) Real-time, in-flow response of the wearable sensor in SWE within the medically relevant pH range; inset: calibration plot (*R*^2^ = 0.970). (e) Signal reversibility in SWE; *V*_app_ = −200 mV.

Once assessed the response of the two-terminal PEDOT:PSS/IrOx Ps pH sensor, the fabrication procedure optimized on glass was implemented using a textile substrate to design a wearable smart dressing for wound healing monitoring. Textile connections were sewed on a medical bandage, and a layer of PEDOT:PSS was screen-printed in between to reproduce the two-terminal geometry. Afterward, IrOx Ps electrodeposition was carried out under the same conditions as described for the glass sensor, and the voltammogram is reported in Figure S12a. The electrochemical characterization (Figure S12b) demonstrates the successful functionalization of the textile substrate. The sensing layer was then coupled to an absorbing material (thickness of around 2 mm) for wound dressings acting as a fluid reservoir and, at the same time, working as a passive pump for the sample fluid. The resulting smart pad is schematically represented in Figure 7a. To best mimic the dynamic wound environment, the performance of the pH sensing dressing was validated in flow analysis (Figure 7b), where an HPLC pump continuously delivers controlled volumes of the pH-variable solutions to the surface of the integrated textile sensor. The samples move across the sensing gauze thanks to the adsorption gradient generated by coupling the different materials. The pump flow was set to 0.05 mL min^–1^ to simulate the exuding rate of a real wound bed.^74^ The smart dressing ability to monitor real-time pH variations within the whole sensor response was assessed by feeding U. B. aliquots at different pH values to the pumping system and the results are reported in Figure 7c. The wearable device shows the expected polynomial response. Considering NCR data, a good match is found among the coefficients of the interpolating curve obtained under flow analysis conditions using the textile sensor (Figure S13) and those calculated for the sensor fabricated on glass (Figure S7). Furthermore, the smart dressing response was studied under flow analysis conditions using simulated wound exudate (SWE) aliquots in the pH range of interest for wound healing monitoring (Figure 7d). As already verified for the glass substrate tested in U. B. (Figure S8), in such a limited pH interval, the sensor response can be approximated to a linear correlation (*R*^2^ = 0.970) and the resulting sensitivity is (54 ± 5) μA pH^–1^, i.e., statistically comparable with the one obtained with the glass substrate. The reversibility of the smart pad response is reported in Figure 7e, demonstrating the capability of the textile sensor to detect random pH variations in SWE.

Under flow conditions, the response time (*t*_90_) of the textile device was calculated as the time required to achieve the 90% of the steady state current following each pH variation in artificial wound exudate. A *t*_90_ of (1.6 ± 0.3) × 10^2^ s was obtained. Moreover, considering the error associated with a pH value estimated from a current signal lying in the centroid of the regression line in Figure 7d,^75^ the smallest detectable pH variation was estimated in 0.2 pH unit.

The operational stability of the fully assembled textile sensor was studied by keeping it immersed in SWE (pH 7.50) for 15 days. The current generated upon application of *V*_app_ = −200 mV was recorded daily for 1 h. The collected data are presented in Figure S14 and allowed us to get information on both drift and stability during the 2-week-long experiment, which reliably simulates a real use of the smart dressing for monitoring chronic wounds. An average current drift of 1% was obtained during each daily measurement, while the % RSD of the mean current recorded throughout the 2 weeks was equal to 3%. Overall, these results confirm the robustness of the smart dressing structure and textiles components. It is worth pointing out that such a variation of the current recorded over 2 weeks translates in a 19% variation of the estimated pH in SWE. This fact reveals that further improvements should be done to effectively apply the smart dressing to a medically relevant environment. Nevertheless, to the best of our knowledge, this is the first time that the long-term stability of a textile sensor is tested during prolonged contact with the sample solution, and therefore, no comparison with other literature reports can be done. Considering their primal stage of development, we believe that our result represents a step forward among the state-of-the-art wearable technologies and suggests that the identification of standardized protocols for the assessment of their analytical performances would be beneficial to the whole research field.

## Conclusions

The combination of a soft, organic semiconductor with a metal oxide potentiometric transducer has been proposed here to design a novel pH sensing material showing promising features for engineering of wearable devices. We demonstrated that the intimate interaction between PEDOT:PSS and IrOx Ps originates a pH sensing mechanism based on electrochemical gating, which has two major consequences.

First, it allows the realization of a two-terminal device that benefits from the robustness of a potentiometric transduction regardless of its essential, chemoresistor-like structure. Second, this configuration makes a solid-state probe compatible with textile substrates and for flow analyses, when provided with a suitable sampling system for the noninvasive collection of biofluids. It is worth noting that under such conditions, where a real-life application was simulated through the realization of a smart wound dressing operating with a small and continuous flow of synthetic exudate, the textile sensor showed no statistically different analytical performances in the pH range relevant to wound healing, i.e., 6–9, with respect to the device fabricated on glass, with metal connections and tested in buffer solutions. Moreover, major advancements are achieved with respect to the state of the art in the pH sensors of textiles, mostly relying on PANi-based working electrodes in a conventional potentiometric setup, and regard both the elimination of the reference electrode and the adaptability to flexible and textile substrates without affecting the sensor reliability. The normalized sensitivity of the two-terminal sensor presented here is almost 1 order of magnitude higher in the medically relevant pH range for wound monitoring than the only example of electrochemically gated pH sensor reported to date^49^ and based on dye-doped PEDOT. To the best of our knowledge, this is the first time that a fully textile pH sensing bandage is validated during a dynamic flow analysis using simulated wound exudate, thus representing a step forward toward the development of smart textiles for biomedical purposes.

## Supporting Information Available

DLS characterization; cyclic voltammograms of a PEDOT:PSS film in U. B.; AFM characterization of a Au film after IrOx Ps electrodeposition; OCP response of a PEDOT:PSS film; effect of *V*_app_ on the two-terminal sensor response; repeatability of the two-terminal sensor in U. B.; reproducibility of the two-terminal sensors in U. B.; approximation of the polynomial response with linear responses; effect of IrOx Ps loading on the sensing performance; two-terminal sensor stability; experimental setup used for the simultaneous recording of the electrochemical potential and current; IrOx Ps electrodeposition on a textile PEDOT:PSS printed electrode and its electrochemical characterization; calibration plot obtained from the in-flow, real-time pH measurement with the smart dressing; and long-term stability test of the smart dressing exposed to SWE (PDF)
