# Supplementary material for: Advanced Wound Dressing for Real-Time pH Monitoring
Source: ACS Sens. 2021 Jun 2;6(6):2366–77. doi: 10.1021/acssensors.1c00552 (PMC8294608; doi:10.1021/acssensors.1c00552)
Supplement: Supplementary file 1 — se1c00552_si_001.pdf [file se1c00552_si_001.pdf]

Advanced wound dressing for real-time pH monitoring

Federica Mariani [1,*], Martina Serafini [1], Isacco Gualandi [1,*], Danilo Arcangeli [1], Francesco Decataldo [2], Luca Possanzini [2], Marta Tessarolo [2], Domenica Tonelli [1], Beatrice Fraboni [2], Erika Scavetta [1]

[1] Dipartimento di Chimica Industriale "Toso Montanari", Università di Bologna, Viale del Risorgimento 4, 40136 Bologna, Italy

[2] Dipartimento di Fisica e Astronomia, Università di Bologna, Viale Berti Pichat 6/2, 40127 Bologna, Italy

Supporting Information

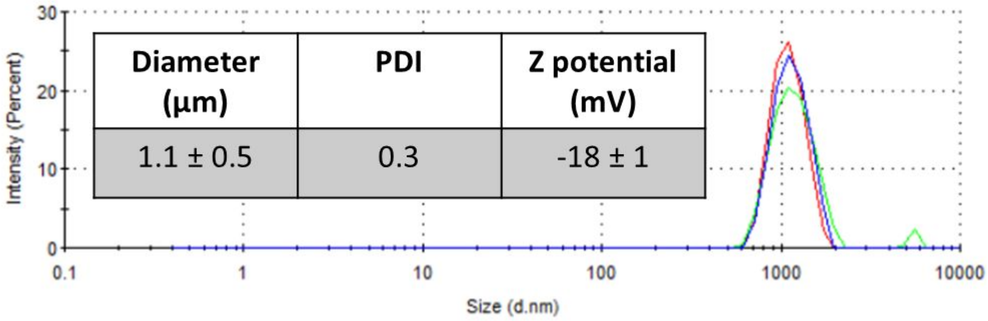

Diameter (µm)	PDI	Z potential (mV)
1.1 ± 0.5	0.3	-18 ± 1

Figure S1. IrOx particles diameter distribution (N = 3) obtained by DLS characterisation. Average diameter, polydispersity index (PDI) and Z potential are reported in the table.

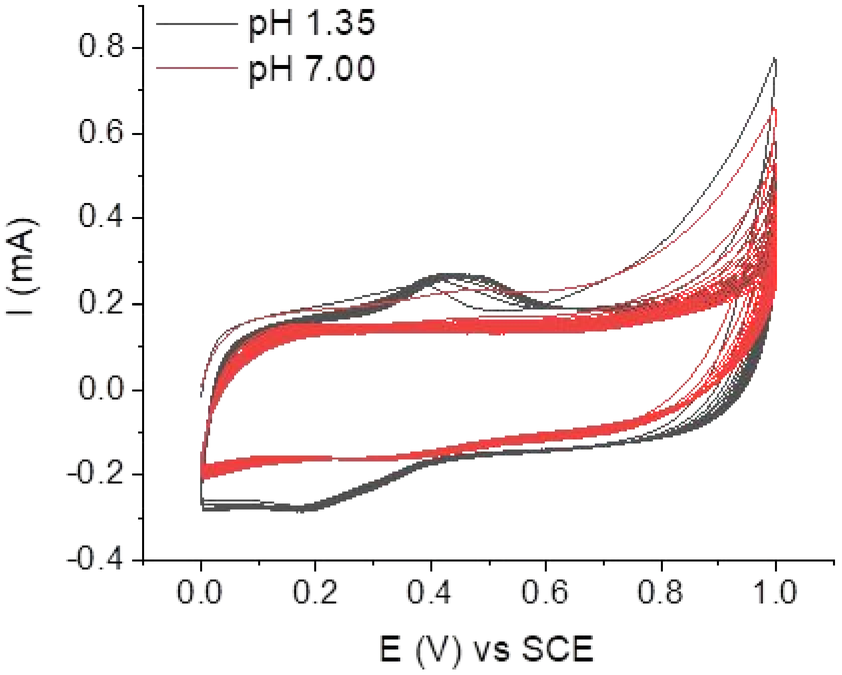

Figure S2. Cyclic voltammograms of a PEDOT:PSS film in U. B. recorded in acidic and neutral pH conditions. Scan rate: 20 mV s^{-1}.

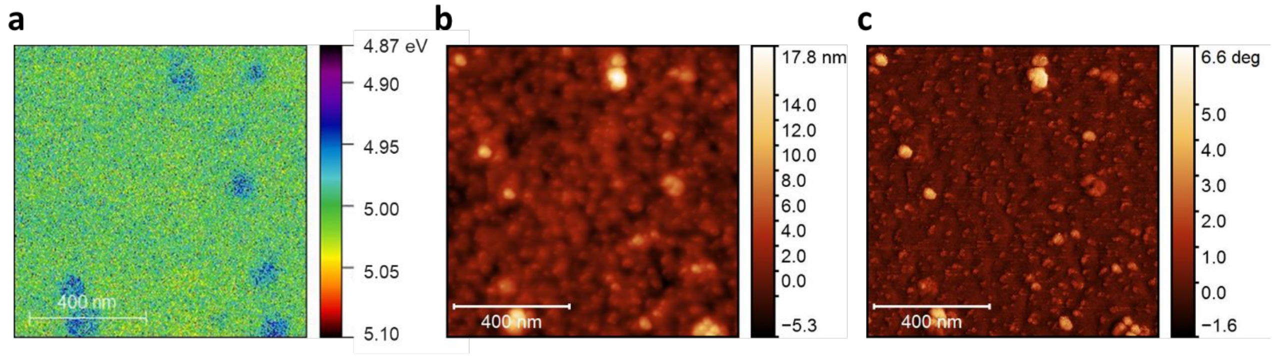

Figure S3. (**a**) KPFM, (**b**) AFM height and (**c**) phase profiles of a Au film after IrOx Ps electrodeposition.

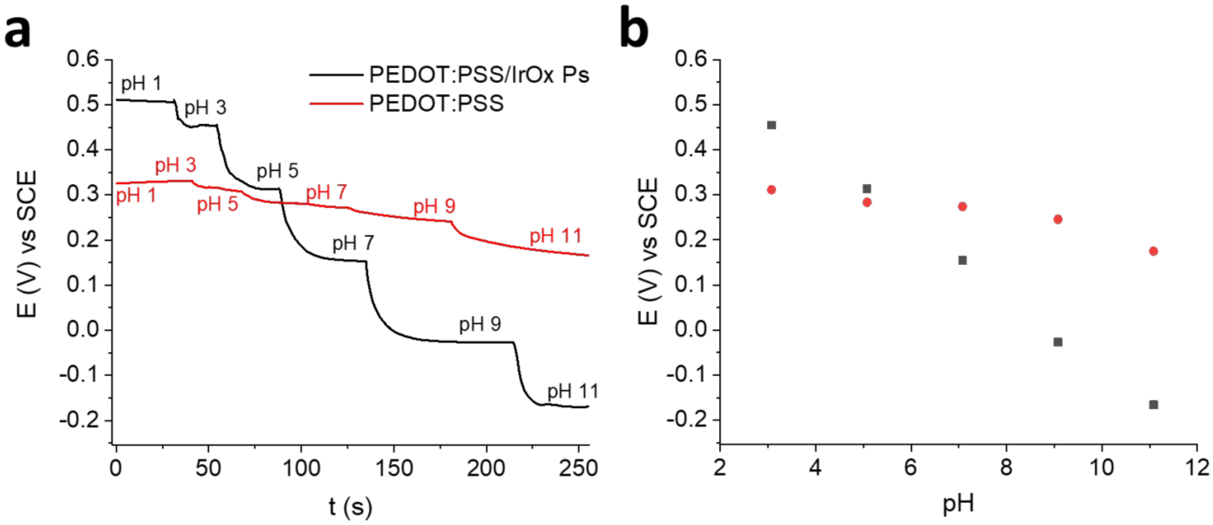

Figure S4. (**a**) OCP responses and (**b**) correspondent E vs pH plots obtained from a PEDOT:PSS film functionalised with IrOx Ps and the pristine PEDOT:PSS film.

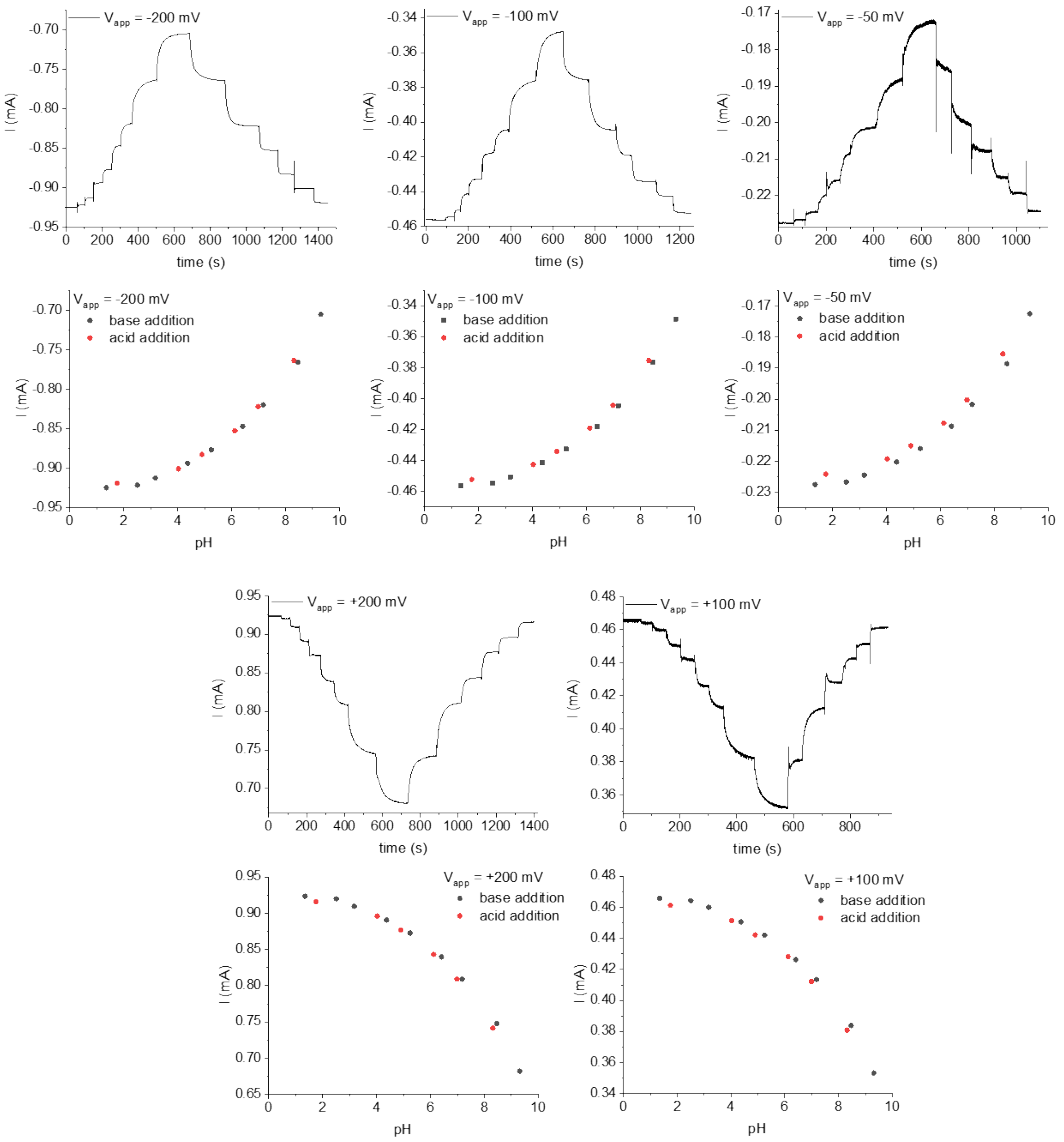

Figure S5. Effect of V_{app} on the two-terminal sensor response.

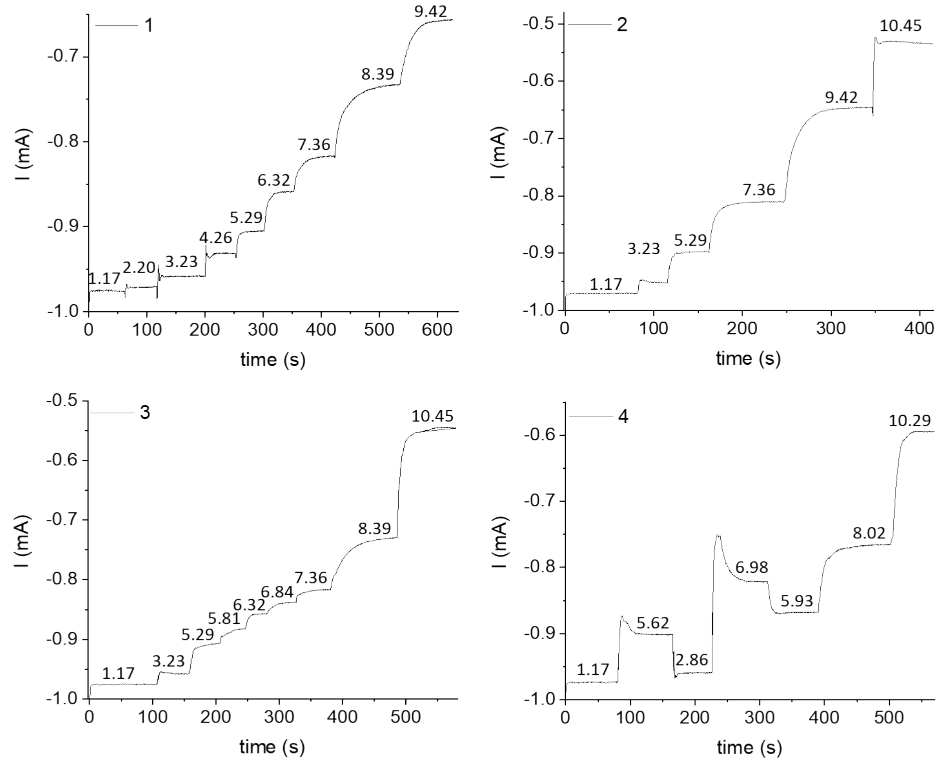

Figure S6. Repeatability of the two-terminal sensor in U. B. Current vs time responses relative to the calibration plots showed in Figure 4 d. V_{app} = -200 mV.

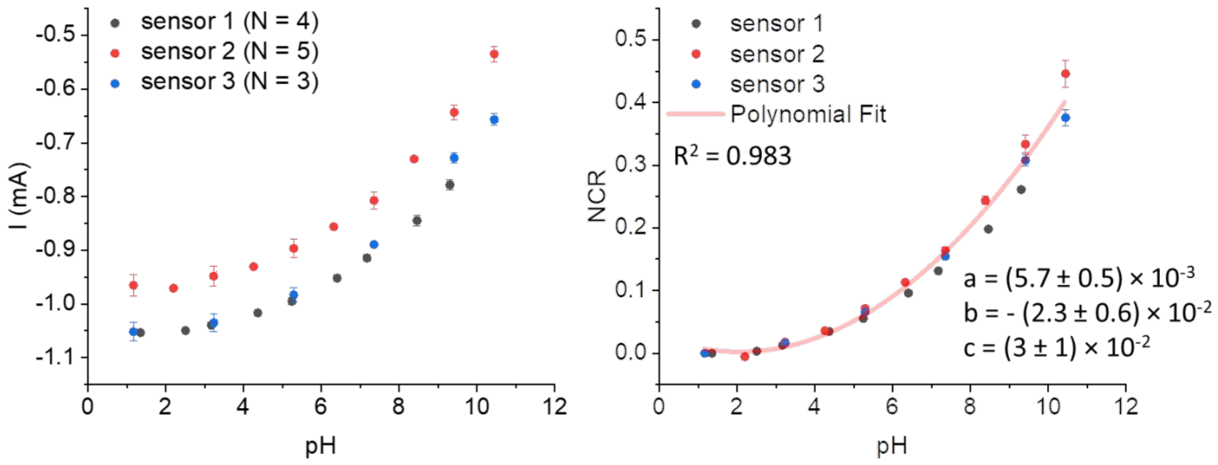

Figure S7. Reproducibility of the two-terminal sensors. Left: current/pH calibration plots; right: normalised current response (NCR)/pH calibration plots. V_{app} = -200 mV.

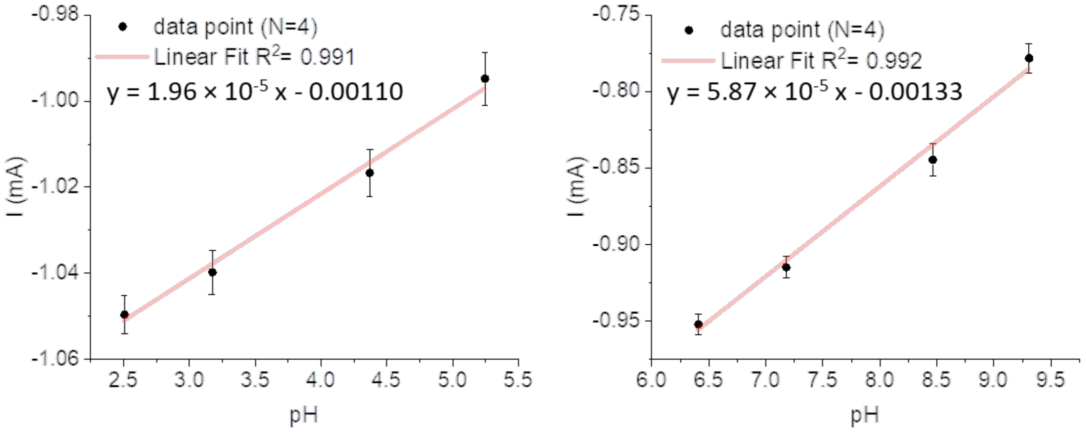

Figure S8. Approximation of the polynomial response with linear responses in acidic (left) and basic (right) environments.

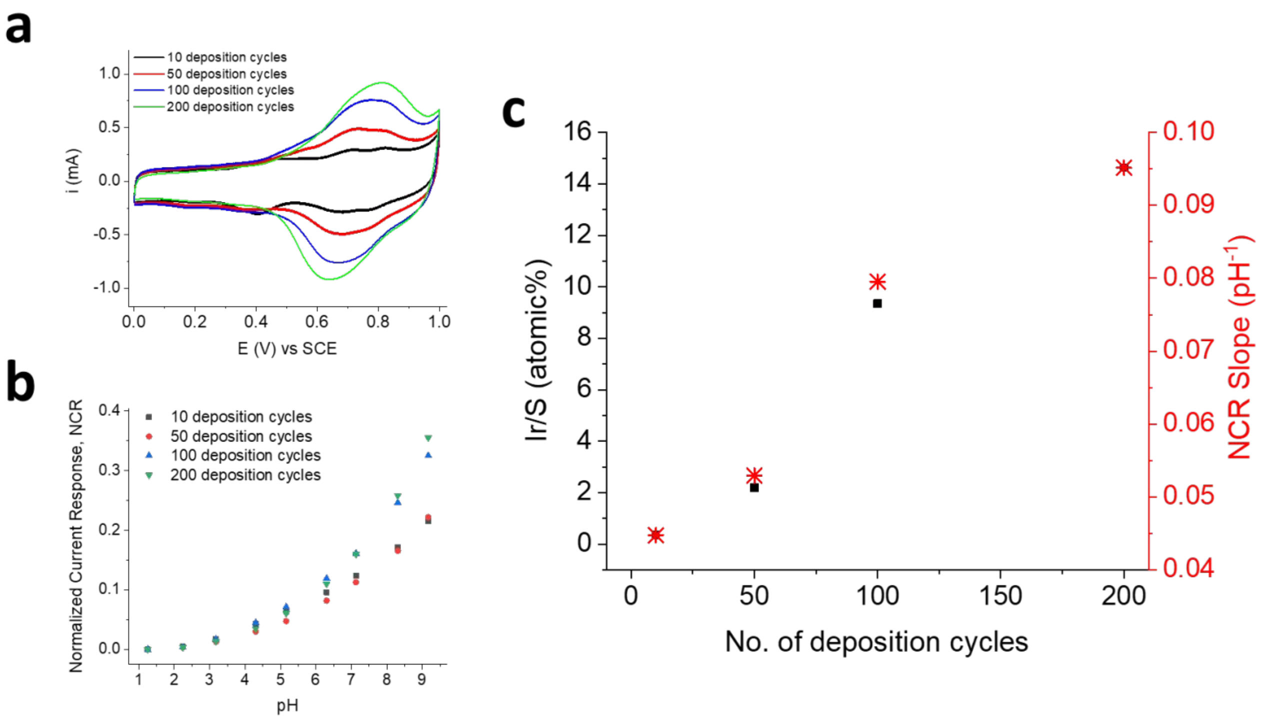

Figure S9. **(a)** Last cycle of the voltammograms recorded during electrochemical depositions using different numbers of cycles. **(b)** Comparison among the normalised current responses (NCR) obtained from the four sensors during pH detection in U.B. V_{app} = -200 mV. **(c)** Plot of Ir/S atomic ratio, calculated from EDS analysis, and NCR sensitivity in the pH range 6.4 – 9.3 as functions of the number of deposition cycles.

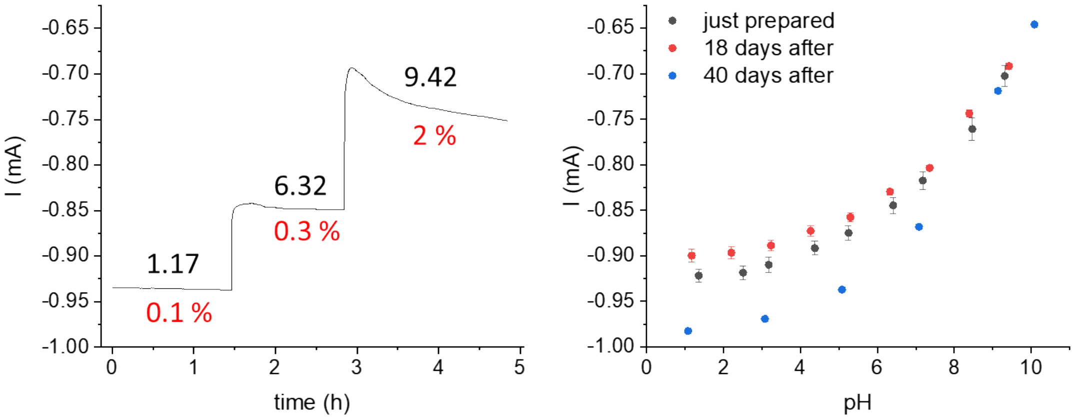

Figure S10. Two-terminal sensor stability during daily use (left) and over a long-term period (right). V_{app} = -200 mV.

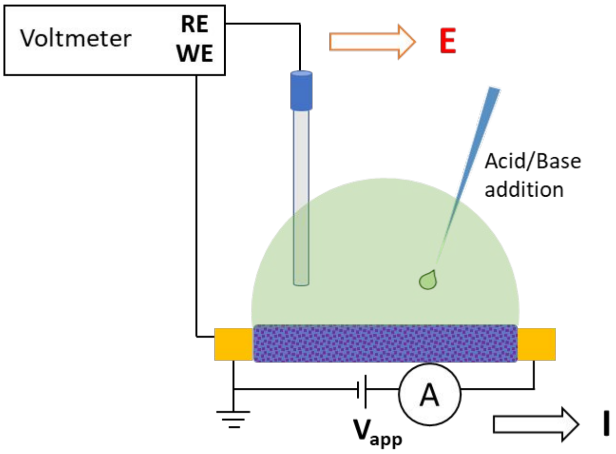

Figure S11. Experimental setup used for the simultaneous recording of the electrochemical potential and current during variations in the pH of the electrolyte solution.

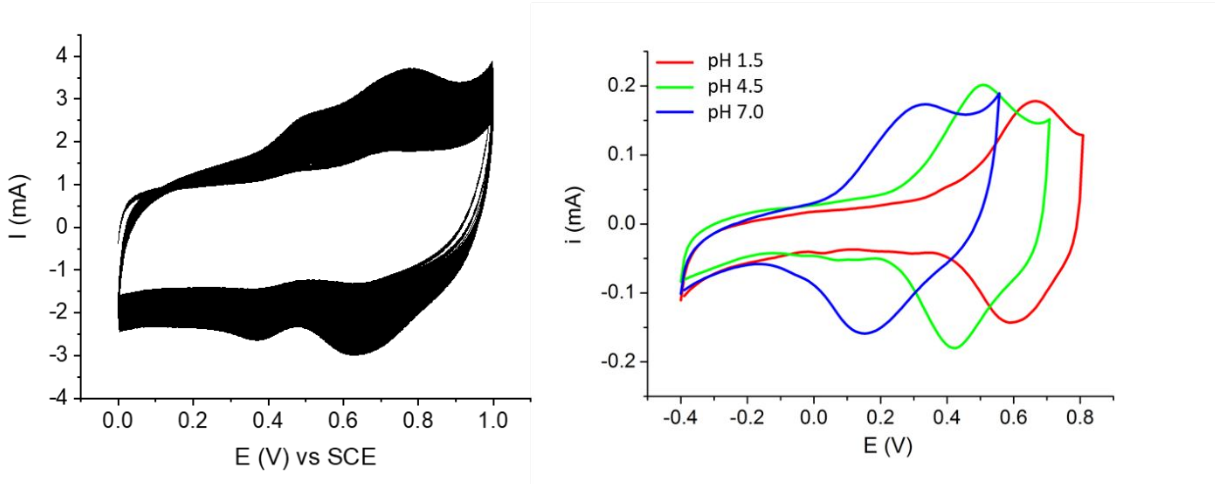

Figure S12. IrOx Ps electrodeposition on a textile PEDOT:PSS printed electrode (left) and cyclic voltammograms recorded from the resulting PEDOT:PSS/IrOx Ps textile electrode in buffers at different pH (right, scan rate 20 mV s^{-1}).

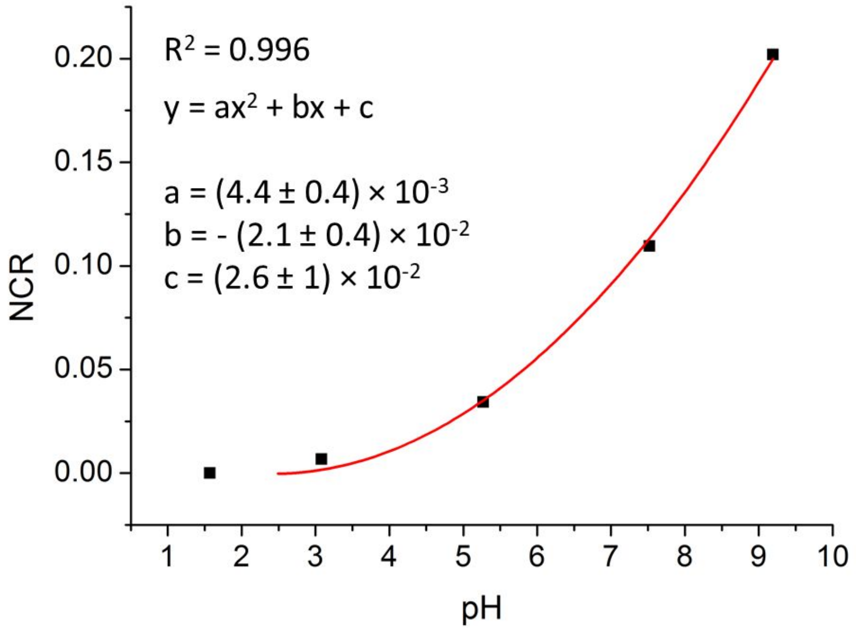

Figure S13. NCR calibration plot obtained from the in-flow, real-time pH measurement with the smart dressing showed in Figure 7 d.

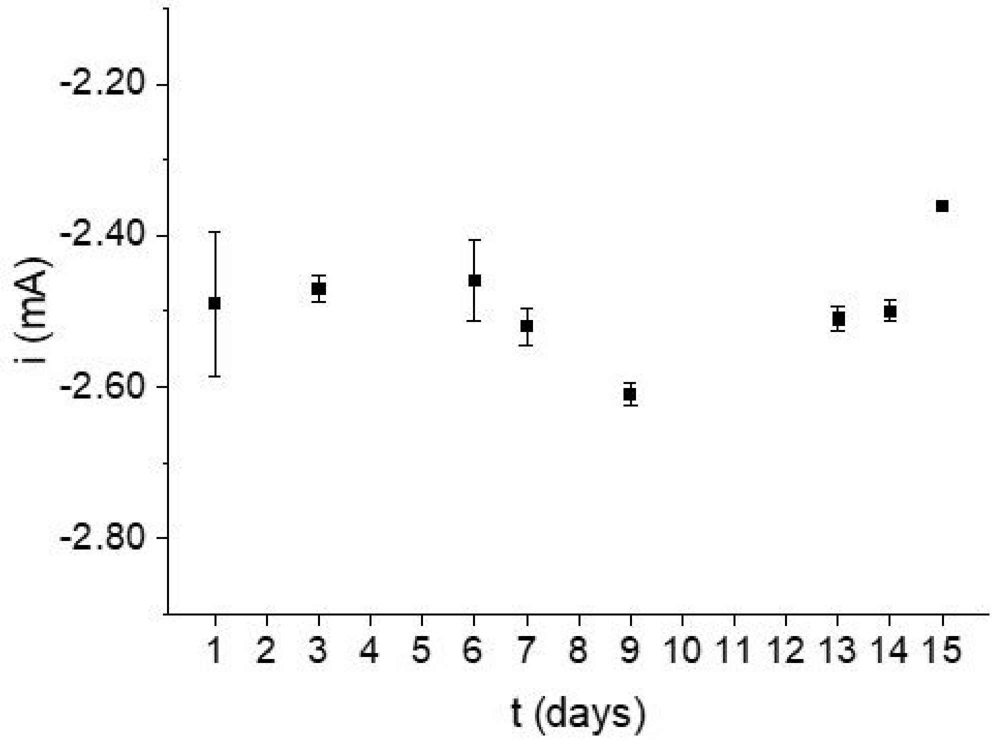

Figure S14. Long-term stability data collected during a two-weeks experiment in which the smart dressing was kept immersed in SWE (pH 7.50). Data points represent the average current value recorded for 1 hour. Error bars represent standard deviations.
